# Yayoi Kusama’s Art and Its Clinical Relevance to Neurovisual Phenomena

**DOI:** 10.7759/cureus.103112

**Published:** 2026-02-06

**Authors:** Lana Liquard, Richard Ho, Enrique Carrazana

**Affiliations:** 1 Clinical Research, University of California San Diego, San Diego, USA; 2 Radiology, Icahn School of Medicine at Mount Sinai, New York, USA; 3 Neurology, University of Hawaii John A. Burns School of Medicine, Honolulu, USA

**Keywords:** hallucinations, neurovisual symptoms, palinopsia, visual perception, visual snow, yayoi kusama

## Abstract

Yayoi Kusama’s art offers a rare and powerful window into how neurological disorders can shape visual perception. Many patients struggle to articulate phenomena, flashing lights, trailing images, and shifting shapes, as these experiences fall outside the ordinary visual vocabulary. Kusama’s lifelong use of endless repetition through polka dots and mirrors parallels descriptions of symptoms such as palinopsia and visual snow. This editorial examines how her own representations of complex visual symptoms can serve as visual analogues, offering clinicians a tool to understand and empathize with patients’ experiences. By interpreting Kusama’s work through a neurological lens, it becomes clear how art can bridge lived perception and clinical observation. The associations between specific artworks and named neurovisual phenomena are intended as educational visual analogies rather than diagnostic attributions.

## Editorial

Yayoi Kusama: Translating neurovisual experience into art

Artists can offer a distinct window into perceptual experience by depicting sensations that are otherwise difficult to describe. In medicine, this perspective can help bridge the gap between subjective sensory experience and clinical interpretation, offering clinicians a clearer sense of how patients navigate their visual phenomena. The purpose of this editorial is to offer clinicians a visual and phenomenological framework for conceptualizing and empathizing with patients who describe complex visual symptoms. Throughout this editorial, references to named neurovisual conditions are used strictly as analogical descriptors of visual experience and should not be interpreted as clinical diagnostic claims.

For many patients living with neurological disorders, describing how the world appears through altered perception is an immense challenge. Flickering lights, afterimages, or distortions of space and size often defy ordinary language, and clinicians frequently rely on patient sketches to glimpse these private visual realities [1,2]. However, what happens when an artist, rather than a patient, devotes a lifetime to translating such visions into art?

Few figures embody this intersection of neurology and creativity as powerfully as Yayoi Kusama (born in 1929 in Matsumoto, Japan). Her work, with fields of polka dots, mirrored rooms, and infinite repetitions, invites the viewer into a world that feels both dazzling and disorienting. Kusama herself has long described her art as an outgrowth of hallucinatory experience; as she wrote, “The main theme of my art, repetitive vision and accumulation, is born from my experience” [3].

In this sense, her oeuvre offers not only a contribution to modern art but also an extraordinary visual lexicon of neurovisual symptoms, providing clinicians and caregivers a tangible reference for understanding patients’ subjective experiences.

From perception to pattern

Kusama’s early life was shadowed by instability and fear. Raised in Matsumoto, Japan, she grew up in a household marked by her mother’s volatile punishments, often for the smallest acts of disobedience, such as playing outdoors, and by the emotional confusion of being forced to spy on her father’s infidelities [4]. These experiences left enduring scars, including an aversion to the male body that would later surface in her explorations of sexuality in art. Amid this turmoil, her garden became her refuge. There, she arranged stones and leaves into secret compositions, seeking in nature a fragile sense of order and safety. Even then, her perception of the world blurred the boundaries between self and environment: she described seeing shimmering fields of dots, hearing voices from flowers, and perceiving auras radiating from objects [5].

The instability of her early life deepened as World War II descended, when, at 13, she was sent to work in a military factory. The ceaseless rhythm of machinery, the wail of sirens, and the omnipresent fear were imprinted on her psyche, subsequently reemerging in the obsessive repetition that became a hallmark of her art. Later, wartime factory labor reinforced an obsessive drive toward order amid chaos.

In 1958, at the age of 27, Kusama left Japan for New York, driven by a determination to escape her restrictive environment and to pursue art on her own terms. New York’s avant-garde scene at the time offered her both freedom and competition, marking a turning point in her life and career. Immersed in the experimental culture of the 1960s, Kusama began to push the limits of painting, sculpture, and performance, befriending artists such as Andy Warhol and Donald Judd. It was during this time that Kusama began experimenting with a diverse range of mediums, including sculpture and performance. These years were crucial in establishing her as a bold, original voice in contemporary art. At the same time, they also coincided with an intensification of her psychological struggles, which she later describes as inseparable from her creative process. For clinicians familiar with palinopsia, the persistence of afterimages, these accounts resonate deeply [6]. Kusama’s own autobiographical accounts describe hallucinatory visual experiences; subsequent associations with specific neurovisual syndromes represent phenomenological analogies proposed by the authors rather than documented clinical diagnoses.

Kusama’s paintings may thus be read not as metaphors but as literal renderings of perceptual disturbance [3,4].

The world multiplied

The following interpretations are intended as illustrative visual analogies to known neurovisual phenomena and do not imply that specific artworks constitute direct evidence of clinically defined syndromes. Kusama’s work is defined by repetition, accumulation, and the dissolution of boundaries between self and environment. Her early paintings, particularly the “Infinity Nets” series that began in the late 1950s, reveal an almost obsessive attention to detail: endless loops of white paint creating fields of rhythmic motion that seem to expand beyond the canvases. These works emerged from a period of intense isolation in New York, where she painted for days on end, sometimes without sleep. The act of repetition became a way to externalize inner tension through a meditative, near-mechanical process [4].

Yayoi Kusama’s “Infinity Nets” series, first exhibited in New York in 1959 at the Brata Gallery, is a foundational work that embodies her personal visual experiences, including palinopsia. These paintings feature countless small, meticulously repeated loops or arcs layered over the canvas, creating a vast, immersive netlike pattern. Kusama described the process as meditative and a way to evoke the infinite, mirroring the persistent and recurring afterimages characteristic of palinopsia [6,7]. The series contrasts with the gestural abstraction dominant at the time, offering a unique feminine perspective of obsessive repetition and infinity. Kusama’s “Infinity Nets” are recognized as a radical reinvention of abstract painting, reflecting her internal visual perceptions rooted in neurological symptoms [5].

Another example of palinopsia in Yayoi Kusama’s work is her installation “Dots Obsession” (1996). This piece features a small, claustrophobic room painted yellow and covered entirely with polka dots of varying sizes on the walls, floor, and ceiling, creating an immersive visual environment where the repetitive dots seem to persist and echo infinitely. The overwhelming repetition and infinite scattering of these dots evoke the persistent afterimages and visual echoes characteristic of palinopsia [6]. Viewers experience a sensation of endless recurrence, reflecting Kusama’s neurological symptoms through spatial and visual immersion.

**Figure 1:**
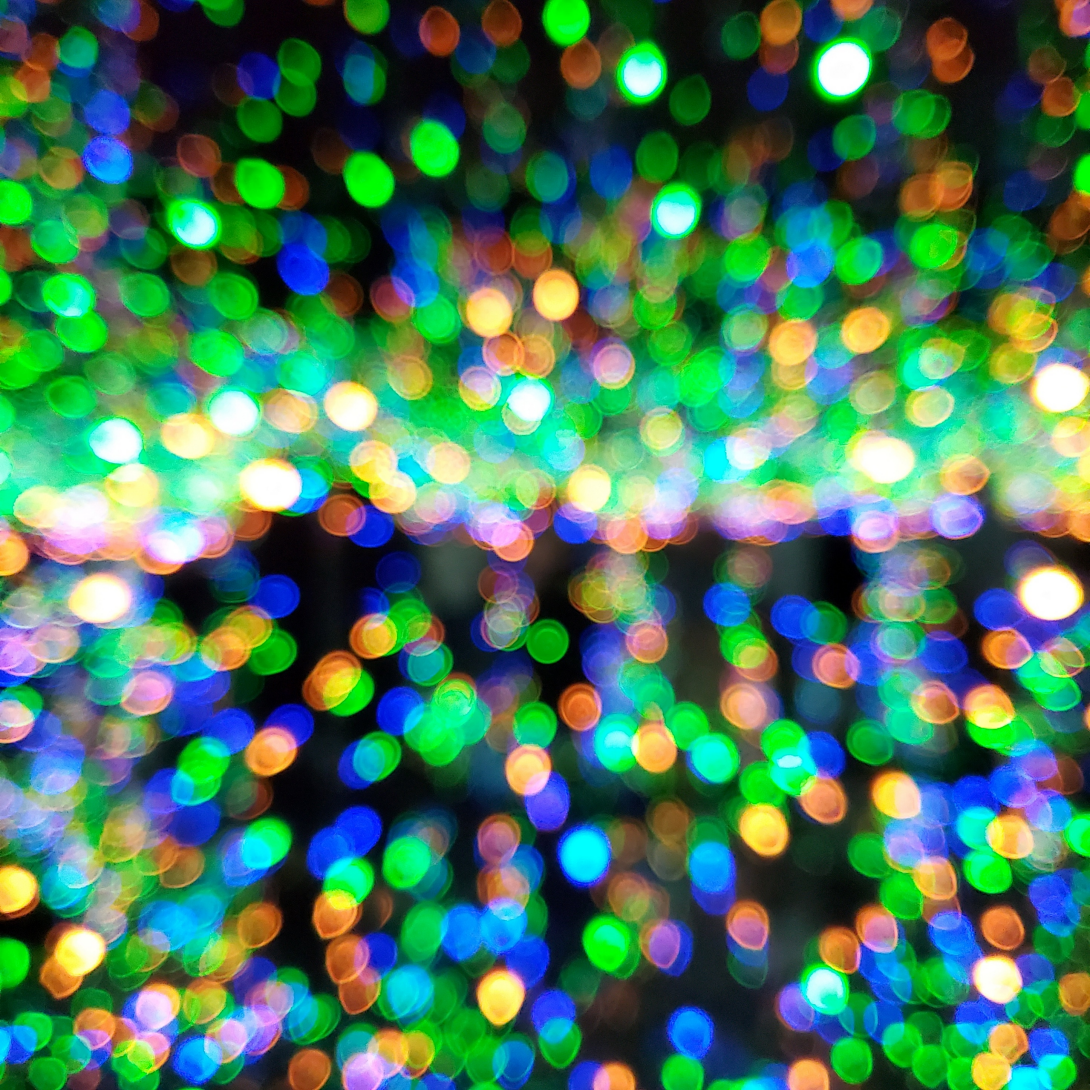
Abstract Simulation of Perceptual Field Distortion, Inspired by Visual Intensity Reminiscent of Yayoi Kusama’s Immersive Installations Photograph by author EC (2025)

As her practice evolved, Kusama began to move beyond the flat surface of the canvas, translating her visual language into three-dimensional space. Her Infinity Mirror Rooms were first conceived in the mid-1960s and continually reimagined throughout her career. They invited viewers into a fully immersive environment where mirrors, lights, and repeated forms seem to multiply infinitely. In works such as “Brilliance of Life” (2012) and “Let’s Survive Forever” (2017), the viewer is surrounded by reflections that stretch infinitely, punctuated by lights that shimmer and pulse in rhythmic constancy [5]. There is no fixed horizon, only unrelenting multiplication of form and light (Figure 1). Kusama is immersing the viewer’s visual fields in faint flickering specks, akin to the persistent brain-based visual static disorder known as “visual snow” [8].

Kusama’s pumpkin sculptures, ranging from miniature to monumental, offer a visual analogy to the perceptual alterations seen in macropsia and micropsia, conditions in which the brain’s processing of size and space becomes unstable. Her enormous yellow pumpkin, installed at the Matsumoto City Museum of Art, for instance, and the smaller versions displayed in mirrored cases or domestic-scale galleries illustrate this oscillation of scale. The same motif, shifting between intimacy and immensity, echoes the way perceptual experience can expand or contract in disorders of visual-spatial integration [9,10].

Across her sculptures, paintings, and installations, Kusama’s motifs, such as polka dots, pumpkins, mirrored spheres, and organic shapes, function as both personal symbols and universal forms. Her vibrant color palette and use of reflective materials amplify the sense of immersion and disorientation, engaging the viewer to confront vastness, repetition, and the loss of fixed perspective. Over time, these visual strategies evolved into a coherent language through which Kusama could articulate her deepest psychological states, translating private sensation into collective experience (Table 1).

**Table 1:** Examples of Neurovisual Symptoms Reflected in Kusama’s Work

Neurovisual symptoms	Definition	Quotation (adapted)	Representative work
Visual snow	Faint flickering specks throughout the visual fields [8]	“When I was a child, I saw hallucinations of lights, auras, and dense fields of dots that engulfed everything, ceilings, walls, and even my own body” [5]	Infinity Mirrored Room-Let’s Survive Forever (2017), Rochester Art Gallery
Photophobia	Sensitivity to light stimuli [6]	“Repetitive vision and accumulation are born from my experience … deriving from my illness since childhood” [5]	Infinity Mirror Rooms (2022), Tate Modern London
Nyctalopia	Impaired night vision [6]	“I covered the canvas with nets, then the table, floor, and my own body … the nets began to expand to infinity” [9]	Infinity Net A (1965), oil on canvas
Macro/microsomatognosia	Altered perceived body size [10]	“She reached to touch the nets she had painted; they seemed to crawl across her skin” [9]	Infinity Net A (1965)
Micropsia/macropsia	Objects appearing smaller or larger [6]	“At age 7, she heard pumpkins and violets talking to her and saw auras around objects” [9]	Matsumoto City Museum of Art (1989)
Teleopsia/pelopsia	Objects appearing farther or nearer [11]	“I saw auras around objects and heard the speech of plants and animals” [4]	Flowers That Speak All About My Heart Given to the Sky and Pumpkin (2018), Ota Fine Arts Tokyo
Lilliputian hallucination	People appearing miniature [6]	“My vision started to flicker, and I saw swirling blue, red, and white configurations” [4]	Infinity Mirrored Room-Filled with the Brilliance of Life (2012), Tate Modern London
Palinopsia	Persistence of afterimage post-stimulus [6]	“She gazed at the pattern of a red flower on a tablecloth and retained its imprint on her retina …. The colorful spots were projected onto everything around her” [12]	Infinity Nets series (1959) and Brata Gallery “Dots Obsession” (1996)
Visual hallucination	Perception without stimulus [7]	“Violets on the tablecloth broke free and crawled over my body” [4]	Infinity Net Series
Perseverative imagery	Repetitive pattern superimposition [6]	“After gazing at a red flower pattern, I saw the entire room and my body covered with the same motif” [4]	Infinity Mirrored Room

Translating a symptom into a symbol

Kusama has written that her hallucinations are not limited to vision. She has felt the very patterns she painted crawling across her skin [9]. Such somatic experiences parallel macrosomatognosia and microsomatognosia, in which one’s body feels unnaturally large or small [10]. Her recurring pumpkins, polka dots, and organic forms often capture oscillations between familiarity and distortion. Quoted descriptions in this section derive from Kusama’s own writings and interviews; the linkage to specific neurological terminology represents interpretive phenomenological framing by the authors.

While never formally diagnosed, Kusama documented anxiety, depression, and what she termed “transient schizophrenia.” She stated, “If it weren’t for art, I would have killed myself a long time ago” [9]. Through her practice, painting became both therapy and testimony, translating torment into texture and symptom into symbol [4].

To stand before Kusama’s work is to glimpse how fragile and elastic human perception can be. In “Let’s Survive Forever” and “Infinity Mirrored Room-Filled with the Brilliance of Life,” light becomes both interference and revelation [8]. Each dot and reflection recall the sensory persistence of visual snow or the trailing of palinopsia [3,11,12].

For neurologists, psychiatrists, and neuroscientists, Kusama’s visual language provides a pedagogical bridge between subjective phenomenology and observable expression (Figure 1).

Seeing through Kusama’s eyes

By transforming complex and often hard-to-explain perceptual experiences into vivid, tangible artworks, Kusama’s oeuvre opens a window of empathy and understanding between patients and clinicians. Her work enhances communication, allowing clinicians to grasp what might otherwise remain elusive in patient descriptions, fostering deeper insight into neurovisual disturbances [2]. In this way, her art becomes a form of neurological storytelling, translating altered perception into a shared aesthetic language that dignifies perceptual disorders by turning private struggles into universal expression [5,9]. Kusama’s installations provide not only a visual metaphor but also a powerful language of empathy: to engage with her work is to momentarily see through the eyes of someone who lives within relentless visual repetition. While this framework is conceptual and illustrative rather than derived from systematic clinical data, it offers a potentially valuable educational bridge between subjective patient experience and clinical interpretation. Future work could explore whether curated visual libraries inspired by Kusama’s motifs may serve as communication tools in clinical settings, aiding history-taking and improving the shared understanding of subjective visual complaints, pending formal validation studies.
